# Phosphorylation of USP27X by GSK3β maintains the stability and oncogenic functions of CBX2

**DOI:** 10.1038/s41419-023-06304-y

**Published:** 2023-11-29

**Authors:** Yushu Xing, Jirimu Ba-tu, Chongyang Dong, Xiaodong Cao, Bing Li, Xin Jia, Yu Juan, Xiaojie Lv, Huiwen Zhang, Na Qin, Wuri Han, Dongfeng Wang, Xiao Qi, Yutong Wang, Xulu Hao, Shuang Zhang, Xiaoli Du, Huanyun Wang, Minjie Wang

**Affiliations:** 1 College of Pharmacy, Inner Mongolia Medical University, Hohhot, Inner Mongolia China; 2 The Center for New Drug Safety Evaluation and Research, Inner Mongolia Medical University, Hohhot, Inner Mongolia China; 3 Medical Innovation Center for Nationalities, Inner Mongolia Medical University, Hohhot, Inner Mongolia China; 4 College of Traditional Chinese Medicine, Inner Mongolia Medical University, Hohhot, Inner Mongolia China; 5 College of Mongolian Medicine, Inner Mongolia Medical University, Hohhot, Inner Mongolia China; 6 Medical Experimental Center of Basic Medical School, Inner Mongolia Medical University, Hohhot, Inner Mongolia China

**Keywords:** Breast cancer, Prognostic markers

## Abstract

Chromobox protein homolog 2 (CBX2) exerts a multifaceted impact on the progression of aggressive cancers. The proteasome-dependent pathway is crucial for modulating CBX2 regulation, while the specific regulatory roles and mechanisms of deubiquitinating enzymes targeting CBX2 remain poorly understood. Mass spectrometry analysis identified ubiquitin-specific peptidase 27X (USP27X) as a deubiquitinating enzyme that targets CBX2. Overexpression of USP27X significantly enhances CBX2 levels by promoting deubiquitination, while deficiency of USP27X leads to CBX2 degradation, thereby inhibiting tumorigenesis. Furthermore, it has been revealed that glycogen synthase kinase 3 beta (GSK3β) can directly bind to and phosphorylate USP27X, thereby enhancing the interaction between USP27X and CBX2 and leading to further stabilization of the CBX2 protein. Clinically, the co-expression of high levels of USP27X and CBX2 in breast cancer tissues is indicative of a poor prognosis for patients with this disease. These findings collectively underscore the critical regulatory role played by USP27X in modulating CBX2, thereby establishing the GSK3β-USP27X-CBX2 axis as a pivotal driver of malignant progression in breast cancer.

## Introduction

Breast cancer (BC) remains a significant global health concern and continues to be the leading cause of cancer-related mortality in women [1]. Among its diverse subtypes, triple-negative breast cancer (TNBC) is characterized by the absence of estrogen receptor (ER), progesterone receptor (PR), and human epidermal growth factor receptor 2 (HER-2) expression [2]. TNBC presents a more aggressive phenotype characterized by heightened invasiveness, increased metastatic potential, greater risk of relapse, and poorer prognosis compared to other BC subtypes [3]. The development and progression of TNBC is driven by critical mutations in key signal transduction pathways including TP53, EGFR, c-KIT, VEGF, and AR that play a pivotal role in its tumorigenesis [4–8]. Given the aggressive nature of TNBC, identifying potential therapeutic targets is crucial for improving patient prognosis and quality of life [9]. Therefore, investigating novel molecular mechanisms involved in the progression of TNBC holds paramount importance in developing effective treatment strategies.

The post-translational modification of proteins known as ubiquitination is a crucial process that has been observed to be dysregulated in several types of cancers, including TNBC [10]. The ubiquitin system is composed of a group of enzymes that covalently attach the 76-amino acid ubiquitin protein to specific target proteins [11]. The enzymes involved in ubiquitination encompass E1 ubiquitin-activating enzymes, E2 ubiquitin-conjugating enzymes, and E3 ubiquitin ligases, as well as a diverse range of deubiquitinating enzymes (DUBs) [12]. DUBs play a significant role in tumorigenesis by removing ubiquitin from substrate proteins through the process of deubiquitylation [13]. Several families of DUBs are present in the human proteome, including UCHs, USPs, OTUs, JAMM, MCPIPs, and MINDYs [14]. The diversity of these DUB families highlights the intricacy of deubiquitylation and its potential involvement in cancer progression. USP27X, a member of the deubiquitinase (DUB) family, plays a critical role in regulating protein ubiquitination and stability [15]. Its oncogenic functions are context-dependent and vary across specific cancer types [16]. For example, USP27X has been implicated in promoting cell proliferation and invasion in hepatocellular carcinoma (HCC) and colorectal cancer (CRC) [17]. Conversely, USP27X has been suggested to exert tumor-suppressive effects in ovarian and lung cancer by inhibiting cell growth and metastasis [18]. Additionally, USP27X has been implicated in modulating the response to anticancer therapies [19]. In glioblastoma, it has been found that USP27X confers resistance to temozolomide, a commonly used chemotherapy drug [20]. On one hand, the downregulation of USP27X in lung carcinoma cells has been demonstrated to enhance their sensitivity to cisplatin therapy. On the other hand, the precise role of this protein in BC progression remains largely unknown [21].

CBX2, a member of the polycomb group (PcG) complex, plays a pivotal role in tumorigenesis across various cancer types [22–28]. It has been implicated as a key player in promoting tumor occurrence and development in gastric carcinoma, colon cancer, and hepatic carcinoma [24, 29]. Interestingly, CBX2 exhibits both tumor suppressive and promoting functions depending on the specific cancer type [30–33]. In glioma, CBX2 inhibits tumor growth; whereas in lung, liver, and colon cancers it promotes tumorigenesis. CBX2 functions as a potent tumor accelerator in BC, promoting tumorigenesis through multiple pathways including the PI3K/AKT signaling pathway, metabolic reprogramming, and intronic DNA methylation, which possibly mainly attribute to regulation of gene expression at chromatin [25, 34–36]. The activation of CBX2 amplifies the pivotal function of the PI3K/AKT signaling pathway in the advancement of BC, as it augments the proliferation, survival, and invasion of BC cells [34]. Additionally, CBX2 assumes a critical role in metabolic reprogramming by stimulating the Warburg effect, which is typified by escalated glycolysis and diminished oxidative phosphorylation. This metabolic shift provides cancer cells with the energy required for rapid growth and proliferation. Additionally, CBX2 induces intronic DNA methylation, an epigenetic modification that can result in aberrant splicing and gene expression, thereby promoting BC progression. Dysregulation of CBX2 in BC is also associated with alterations in other epigenetic marks, including histone modifications and chromatin remodeling. In aggregate, CBX2 functions as a tumor accelerator in BC through multiple pathways. Given its multifaceted role in tumorigenesis, CBX2 represents a promising therapeutic target for the development of novel therapies for BC. Further investigation into the underlying mechanisms of CBX2 dysregulation in BC is imperative for a comprehensive understanding of its role in the disease and identification of potential therapeutic interventions.

Phosphorylation and deubiquitination are two indispensable post-translational modifications that exert pivotal roles in regulating protein function and stability [37]. Phosphorylation involves the covalent attachment of a phosphate group to a protein, while deubiquitination entails the enzymatic cleavage of ubiquitin molecules from a substrate [38]. These two processes are intricately interconnected and critically involved in the precise modulation of diverse cellular processes. Phosphorylation has been shown to modulate deubiquitination through multiple lines of evidence [39–41]. For example, phosphorylation of specific substrates can prevent their degradation via the ubiquitin-proteasome pathway and increase protein stability [42]. This phenomenon has been observed in various proteins, such as p53, c-Jun, and NF-κB [43–45]. Phosphorylation can either inhibit the activity of certain ubiquitin ligases or enhance the function of deubiquitinases. Moreover, phosphorylation can regulate deubiquitination by directly affecting the activity or subcellular localization of certain deubiquitinases. For instance, USP7 is an exemplar that can be phosphorylated at multiple sites by CK2 to strengthen its interaction with substrates and enhance its deubiquitination activity [45]. Similarly, the activity of deubiquitinase USP14 is regulated by phosphorylation through protein kinase CK2 [46]. Phosphorylation at specific sites on USP14 enhances its activity and increases protein stability. Additionally, phosphorylation and deubiquitination can synergistically regulate protein function. For example, deubiquitinase USP15 interacts with and deubiquitinates kinase RIPK1, leading to its stabilization and activation of downstream signaling pathways [47]. This interaction between USP15 and RIPK1 is regulated by phosphorylation of specific sites on USP15, which enhances its binding to RIPK1 and promotes the stabilization of RIPK1. The interplay between phosphorylation and deubiquitination is complex and multifaceted [48]. In addition, the ubiquitin-specific protease (USP7) associates with related complex and deubiquitinates its CBX2, while loss of USP7-mediated deubiquitination results in reduced co-immunoprecipitation of CBX2 [49]. Although the precise mechanisms of this interaction remain to be fully elucidated, it is evident that these two post-translational modifications are closely linked and play pivotal roles in regulating protein function and stability.

Our findings collectively suggest that USP27X plays a critical role in regulating CBX2, and the GSK3β-USP27X-CBX2 axis is pivotal in promoting malignant progression of BC. These results provide novel insights into the regulation of CBX2 and have significant implications for developing innovative therapeutic strategies targeting aggressive forms of BC.

## Results

### USP27X interacts with CBX2

**Fig. 1:**
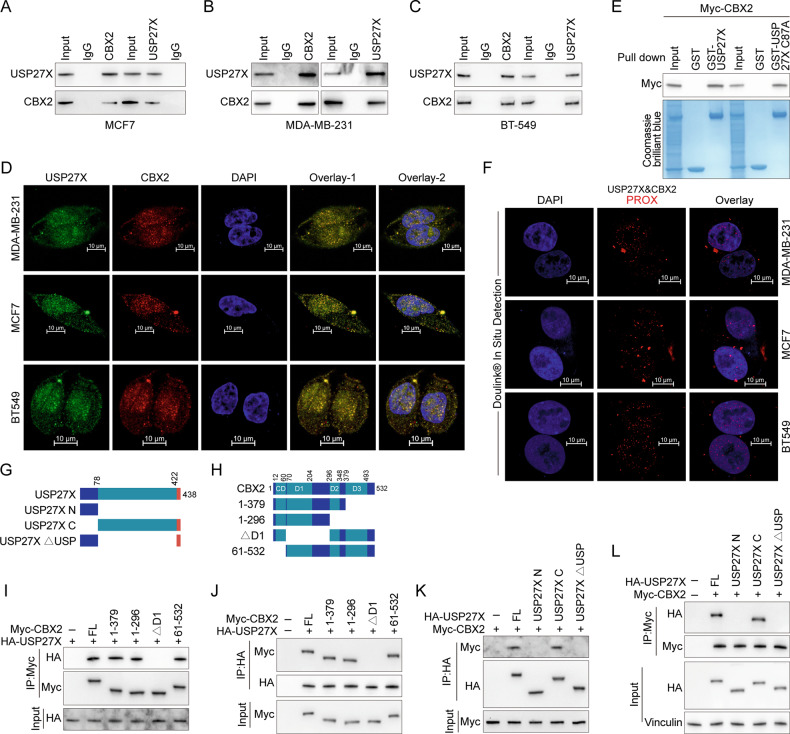
USP27X interacts with CBX2. **A**–**C** Cell lysates from MCF7 (**A**), MDA-MB-231 (**B**), and BT-549 (**C**) were analyzed by IP using antibodies against USP27X and CBX2, then subjected to IB analysis. IgG was used as the isotype control. **D** Triple immunofluorescence (IF) staining of USP27X(green), CBX2(red), and nuclei (DAPI, blue) was performed in MCF7, MDA-MB-231, and BT-549 cells. Scale bar, 10 μm. **E** Purified Myc-CBX2 was incubated with GST, GST-USP27X or GST-USP27X C87A coupled to glutathione-Sepharose beads. Proteins retained on Sepharose were then subjected to IB with indicated antibodies. Recombinant GST-USP27X and GST-USP27X C87A were purified from bacteria and analyzed by SDS-PAGE and Coomassie blue staining. **F** Representative images show merged PLA and nuclear (DAPI) channels from PLA experiments. In situ PLA between endogenous USP27X and CBX2. Each red dot represents detection of the USP27X-CBX2 interaction complex. Scale bar, 10 μm. **G**, **H** Schematic representation of HA-tagged full-length (FL) USP27X (**G**), Myc-tagged FL CBX2 (**H**), and their various deletion mutants. **I**, **J** HEK-293T cells were cotransfected with HA-USP27X and Myc-tagged FL CBX2 or its deletion mutants and anti-Myc or HA-coupled magnetic beads for IP analysis of cell lysates followed by IB analysis with HA and Myc antibodies. **K**, **L** HEK-293T cells were cotransfected with Myc-CBX2 and HA-tagged FL USP27X or its deletion mutants and anti-Myc or HA-coupled magnetic beads for IP analysis of cell lysates followed by IB analysis with HA and Myc antibodies. For all panels, data are representative results of three independent experiments.

The regulatory function of USP27X has been implicated in multiple types of tumor progression, with our research focusing on its potential involvement in the development of BC. Our analysis of the TCGA database identified an abnormally elevated expression level of USP27X within this context (Supplementary Fig. 1A). To further investigate this phenomenon, we established stable BT549 cell lines that express USP27X and utilized mass spectrometry techniques to identify associated immunoprecipitates (Supplementary Fig. 1B). Through functional analysis of potential downstream target proteins, CBX2 was initially identified as one of the top 10 candidates (ranked by the number of specific peptides identified) (Supplementary Fig. 1B). Subsequently, the interaction between USP27X and CBX2 was validated in HEK-293T cells (Supplementary Fig. 1C). Similarly, a physical association between endogenous USP27X and CBX2 proteins was validated in MCF7, MDA-MB-231 and BT549 (Fig. 1A–C). Immunofluorescence co-localization experiments revealed a significant overlap between USP27X and CBX2 within the nuclei of BC cells (MDA-MB-231, MCF7, and BT549), with some co-distribution observed in the cytoplasm (Fig. 1D). The interaction between exogenous USP27X and CBX2 was also verified by immunofluorescence assay in HEK-293T cells (Supplementary Fig. 1D). To validate the direct nature of their interaction, we performed GST-pulldown (Fig. 1E) and proximity ligation assays. Furthermore, we observed PLA signaling in the nucleus and cytoplasm, providing additional evidence for a direct interaction between endogenous USP27X and CBX2 (Fig. 1F). The PLA signal for a direct interaction between exogenous USP27X and CBX2 was detected in HEK-293T cells (Supplementary Fig. 1E). By analyzing truncated mutants of USP27X and CBX2, we further investigated the minimal regions necessary for their interaction (Fig. 1G, H). Our findings indicate that USP27X truncating mutants lacking the USP domain do not bind CBX2, and that absence of the D1 domain in CBX2 disrupts the binding between USP27X and CBX2 (Fig. 1I–L). Therefore, the interaction between USP27X and CBX2 requires the presence of the USP domain in USP27X and the D1 domain in CBX2. Taken together, these results strongly suggest that CBX2 is indeed an interacting partner of USP27X.

### USP27X enhances CBX2 protein stability

**Fig. 2:**
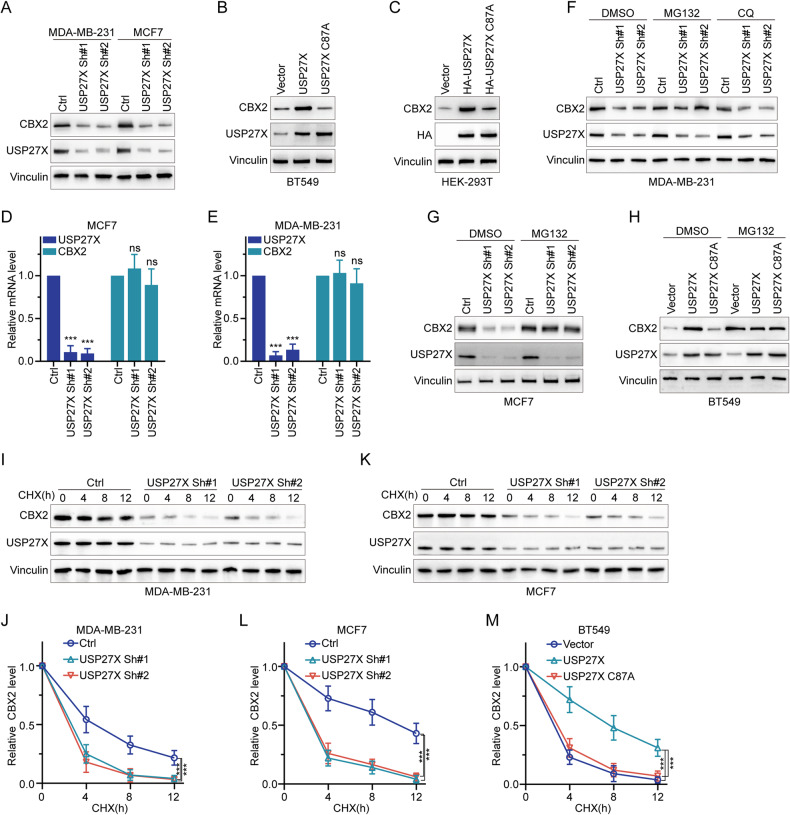
USP27X enhances CBX2 protein stability. **A** USP27X was knocked down in MCF7 and MDA-MB-231 cells by two independent shRNAs. CBX2 protein expression level was detected by IB analysis. **B**, **C** USP27X WT or C87A mutants were overexpressed in BT549 (**B**) and HEK-293T cells (**C**) The level of CBX2 protein was analyzed. **D**, **E** qRT-PCR analysis of CBX2 mRNA expression in MCF7 (**D**) and MDA-MB-231 (**E**) cell lines depleted of USP27X. **F** MDA-MB-231 cells transfected with 2 independent USP27X shRNAs were treated with or without the proteasome inhibitor MG132 (20 μM, 8 h) or CQ (20 μM, 12 h), and then analyzed for USP27X and CBX2. **G** MCF7 cells transfected with two independent USP27X shRNAs were treated with or without the proteasome inhibitor MG132 (20 μM, 8 h), and then analyzed for USP27X and CBX2. **H** BT549 cells transfected with USP27X WT or C87A mutants were treated with or without the proteasome inhibitor MG132 (20 μM, 8 h), and then analyzed for USP27X and CBX2. **I**–**L** MDA-MB-231 (**I**) and MCF7 (**K**) cells stably expressing control shRNA or USP27X shRNA were treated with 100 μg/ml CHX, harvested at the indicated times, and treated with IB with anti-CBX2 and USP27X antibodies. Quantification of CBX2 levels relative to Vinculin is shown. **J** Analyzed results of relative CBX2 level in MDA-MB-231. **L** Analyzed results of relative CBX2 level in MCF7. **M** BT549 cells transfected with USP27X WT or C87A mutants were treated with 100 μg/ml CHX, harvested at the indicated times, and treated with IB with anti-CBX2 and USP27X antibodies. Quantification of CBX2 levels relative to Vinculin is shown. Data are represented as mean ± SD of 3 independent experiments. ***P < 0.001, one-way ANOVA with Dunnett’s post test (**D**, **E**, **J**, **L**, **M**).

The interaction between USP27X and CBX2 suggests that USP27X may act as a deubiquitinating enzyme targeting the degradation of CBX2 protein. To confirm this hypothesis, we observed a reduction in CBX2 expression upon knockdown of USP27X in BC cells (Fig. 2A and Supplementary Fig. 2A–C), while overexpression of wild-type but not catalytically inactive mutant USP27X C87A (a catalytically inactive point-mutant) resulted in an increase in CBX2 expression in BT549 and HEK-293T cells (Fig. 2B, C). The knockdown or overexpression of USP27X, as determined by qRT-PCR, did not exert any effect on the mRNA level of CBX2 (Fig. 2D, E). In eukaryotic cells, the autophagy-lysosomal pathway and ubiquitin-proteasome pathway are two major mechanisms that play crucial roles in maintaining cellular homeostasis, ensuring protein quality control, and regulating cellular responses to stress conditions. Subsequently, we investigated whether USP27X-mediated regulation of CBX2 is contingent upon proteasomal activity. As expected, treatment with the proteasome inhibitor MG132 effectively restored the decreased levels of CBX2 induced by USP27X knockdown, while inhibition of autophagy-lysosomal pathway with CQ failed to exhibit such a restorative effect (Fig. 2F). Similarly, MG132 counteracted the upregulation of CBX2 caused by USP27X overexpression (Fig. 2G, H). These findings collectively indicate that the regulation of CBX2 at the protein level by USP27X is dependent on both proteasome-mediated degradation and its deubiquitinating enzyme activity. To demonstrate the impact of USP27X on CBX2 stability, we employed CHX to inhibit protein synthesis and assessed changes in CBX2 protein levels upon interference with USP27X expression. The current investigation demonstrates that the downregulation of USP27X expression in MDA-MB-231 and MCF7 cells leads to a marked decrease in CBX2 protein stability (Fig. 2I–L). Conversely, the upregulation of USP27X in BT549 cells, but not in the C87A mutant, results in a significant increase in CBX2 protein stability (Fig. 2M and Supplementary Fig. 2D). Taken together, these findings provide evidence for the selective amplification of CBX2 protein stability by USP27X.

### USP27X deubiquitinates CBX2

**Fig. 3:**
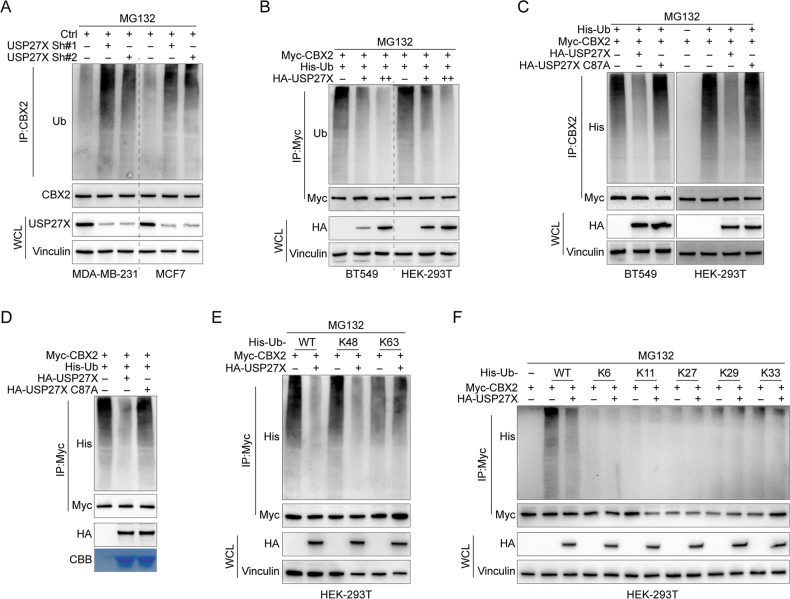
USP27X deubiquitinates CBX2. **A** MCF7 and MDA-MB-231 cells were transfected with 2 independent USP27X shRNAs. Cell lysates were subjected to IP treatment with CBX2 antibodies followed by IB treatment with Ub and CBX2 antibodies. Cells were treated with 20 μM MG132 for 8 h before harvesting. **B** HEK-293T and BT549 cells were transfected with the indicated constructs were treated with MG132 for 8 h before collection. The whole-cell lysates were subjected to IP with Myc antibody and IB analysis with anti-Ub antibody to detect ubiquitylated CBX2. **C** HEK-293T and BT549 cells were cotransfected with Myc-CBX2, His-Ub, and HA-USP27X WT or USP27X C87A, and cell lysates were subjected to IP with Myc followed by IB with antibodies against Myc, HA and His. Cells were treated with 20 μM MG132 for 8 h before harvesting. **D** Ubiquitylated Myc-CBX2 was incubated with HA-USP27X WT or HA-USP27X C87A coupled to glutathione-Sepharose beads. Myc-CBX2 was subjected to IP with Myc beads followed by IB with antibodies against HA and His. Purified HA-USP27X or HA-USP27X C87A was analyzed by SDS-PAGE and Coomassie blue staining. **E** HEK-293T cells were cotransfected with Myc-CBX2, HA-USP27X, and the indicated His-Ub Lys48-only (K48), or Lys63-only plasmids (K63), and then the CBX2 ubiquitylation linkage was analyzed. **F** HEK-293T cells were cotransfected with Myc-CBX2, HA-USP27X, and the indicated His-Ub Lys6-only (K6), Lys11-only (K11), Lys27-only (K27), Lys29-only (K29), or Lys33-only plasmids (K33), and then the CBX2 ubiquitylation linkage was analyzed. For all panels, data are representative results of three independent experiments.

The deubiquitination activity of USP27X on CBX2 was investigated by observing the impact of USP27X knockdown and overexpression on endogenous and exogenous CBX2 ubiquitination levels in MDA-MB-231, MCF7, HEK-293T, and BT549 cells. The results showed that USP27X knockdown increased endogenous CBX2 ubiquitination, while USP27X overexpression decreased CBX2 ubiquitination levels (Fig. 3A, B). Additionally, transfection of HA-USP27X in HEK-293T and BT549 cells resulted in a decrease in the ubiquitination level of exogenous CBX2, while overexpression of USP27X C87A did not exhibit any effect on the ubiquitination level of CBX2 (Fig. 3C). These findings suggest that USP27X is involved in the regulation of CBX2 ubiquitination. To confirm CBX2 as a direct deubiquitinating substrate for USP27X, we conducted a co-incubation experiment using purified HA-USP27X or HA-USP27X C87A under cell-free conditions with polyubiquitinated CBX2. Our results demonstrate that purified HA-USP27X specifically removes the polyubiquitin chains from CBX2 in vitro, while USP27X C87A lacks this ability (Fig. 3D). This implies that USP27X stabilizes CBX2 by directly deubiquitinating it. In fact, the two primary forms of polyubiquitinated chains Lys48 bond and Lys63 bond have been extensively investigated in protein ubiquitination. Lys48-linked ubiquitin chains represent the prototypical form of ubiquitin chains that are associated with protein degradation via the proteasome. By serving as a signal for substrate recruitment to the proteasome, lys48-linked polyubiquitin chains facilitate their unfolding and targeted degradation within the core particle. This pathway plays a crucial role in maintaining protein homeostasis, regulating cell cycle progression, eliminating misfolded or damaged proteins, and modulating levels of short-lived regulatory proteins. Lys63-linked ubiquitin chains possess diverse functions beyond proteasomal degradation, as compared to Lys48-linked ubiquitin chains. This is due to the covalent attachment of the Lys63 residue of one ubiquitin molecule to the Lys63 residue of the next molecule in the chain. The essentiality of Lys63-linked polyubiquitination is evident in several cellular processes such as DNA repair, signal transduction, protein transport, and immune response [50]. Therefore, our research objective was to identify the specific type of polyubiquitin modification on CBX2 protein that is targeted by USP27X. As depicted in Fig. 3E, the deubiquitinating enzyme USP27X efficiently disassembles Lys48-linked polyubiquitination of CBX2 without significantly affecting non-degradable Lys63-linked polyubiquitination. Furthermore, the polyubiquitination of CBX2 through Lys6, Lys11, Lys27, Lys29, and Lys33 linkages exhibits minimal response to USP27X (Fig. 3F). These findings suggest that USP27X is a genuine deubiquitinating enzyme that specifically targets the deubiquitination of CBX2 protein.

### USP27X promotes the proliferation, invasion, and metastasis of BC cells by up-regulating CBX2

**Fig. 4:**
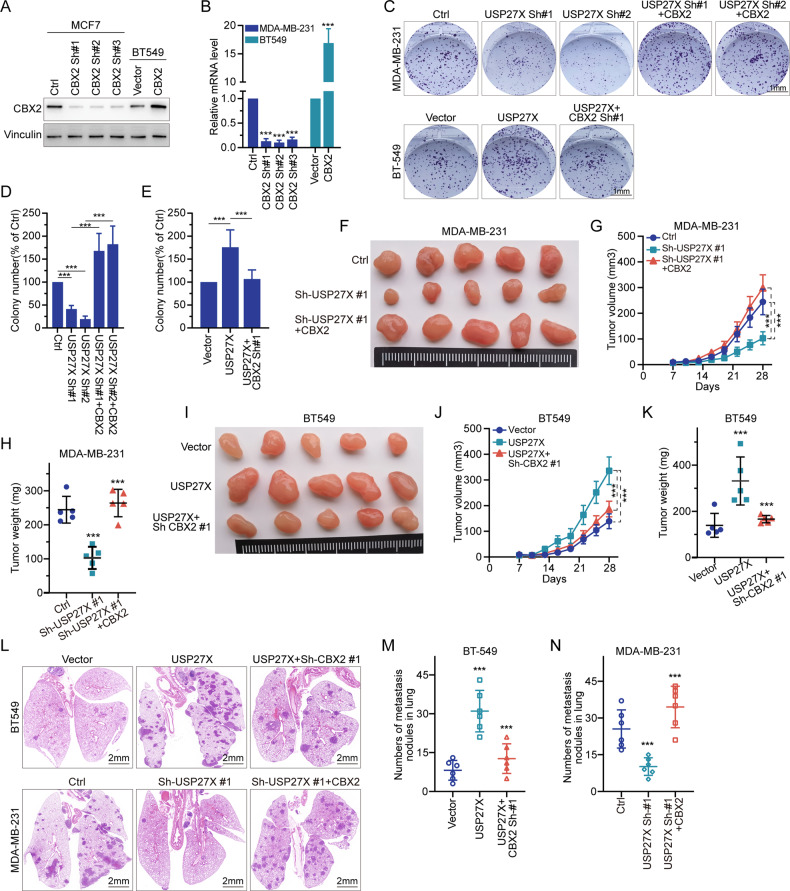
USP27X promotes the proliferation, invasion and metastasis of BC cells by up-regulating CBX2. **A**, **B** In MCF7 cells, three independent shRNAs were used to knock down CBX2. CBX2 was overexpressed in BT549. **A** CBX2 protein expression level was detected by IB analysis, and (**B**) CBX2 mRNA level was detected by qRT-PCR analysis. **C** USP27X was knocked down in CBX2-overexpressing MDA-MB-231 cells. CBX2 was knocked down in BT549 cells overexpressing USP27X. Cell growth was examined by colony formation. **D** Cell treatment with ctrl, two USP27X shRNA (1&2) and USP27X shRNA (1&2) + overexpressed CBX2, leads to a significant decrease and increase of the colony number. **E** Cell treatment with vector, overexpressed USP27X and overexpressed USP27X + CBX2 shRNA leads to a significant increase and decrease. **F** The indicated cells were subcutaneously injected (1 × 10^6^ cells per mouse) into nude mice (*n* = 5) of MDA-MB-231. Tumor growth curve (**G**) and weight (**H**) were analyzed. **I** The indicated cells were subcutaneously injected (1 × 10^6^ cells per mouse) into nude mice (*n* = 5) of BT549. Tumor growth curve (**J**) and weight (**K**) were analyzed. **L**–**N** The indicated cells were injected into the mammary fat pad of female NOD-SCID mice (5-6 weeks old, *n* = 6) of BT549 and MDA-MB-231 (**L**). The number of pulmonary metastatic nodules was counted and quantified of BT549 (**M**) and MDA-MB-231 (**N**). Data are represented as mean ± SD of three independent experiments. ***P < 0.001, 1-way ANOVA with Dunnett’s post test (**B**, **D**, **E**, **G**, **H**, **J**, **K**, **M**, **N**).

In the field of oncology, the CBX2 gene has garnered significant attention due to its involvement in tumorigenesis [51]. The present study explores the impact of USP27X on BC progression by regulating CBX2. CBX2 is known to modulate genes that regulate cell cycle progression, apoptosis, and DNA damage response, leading to uncontrolled proliferation of cancer cells. Additionally, CBX2 promotes epithelial-mesenchymal transition (EMT), which is associated with increased invasive and metastatic potential of cancer cells [52]. The results of this study indicate that knockdown of USP27X in MDA-MB-231 cells inhibits proliferation, whereas overexpression of USP27X in BT549 cells promotes tumor growth. Furthermore, these effects were reversed upon modulation of CBX2 expression. Furthermore, knockdown of USP27X resulted in the inhibition of MCF7 invasion and metastasis, whereas overexpression of USP27X facilitated invasion and metastasis in BT549. Notably, this effect was reversed by either overexpressing or knocking down CBX2 (Fig. 4A–E and Supplementary Fig. 3A–C). In vivo experiments also demonstrated that knockdown of USP27X suppressed tumor proliferation in xenograft models (Fig. 4F–H), while overexpression of USP27X promoted tumor proliferation in BT549 xenograft models (Fig. 4I–K). Again, this effect was counteracted by manipulating CBX2 expression levels. Knockdown of USP27X was found to decrease the incidence of lung metastases from BC cells, while overexpression of USP27X had the opposite effect (Fig. 4L). The aforementioned phenomenon was found to be reversed through either overexpression or knockdown of CBX2, as depicted in Fig. 4L–N. These results suggest that USP27X facilitates the growth and metastasis of BC cells by up-regulating CBX2, both in vivo and in vitro.

### GSK3β enhances the stability of CBX2 protein by phosphorylating USP27X

**Fig. 5:**
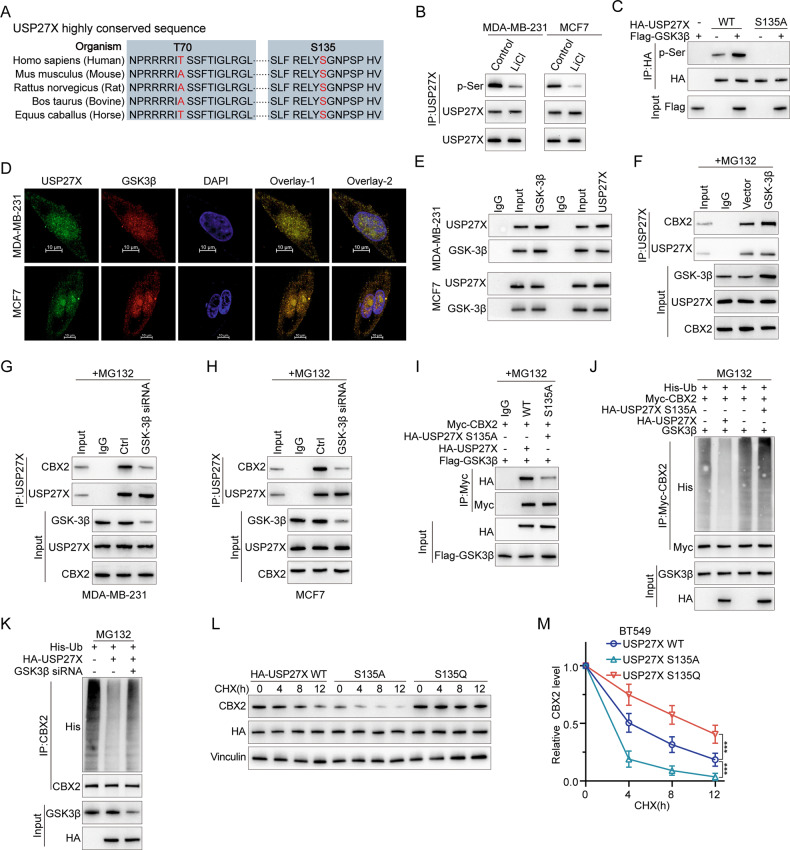
GSK3β enhances the stability of CBX2 protein by phosphorylating USP27X. **A** Amino acid sequence conservation in different species of the motif in USP27X targeted by GSK3β. **B** MDA-MB-231 or MCF7 cells were treated with lithium chloride for 6 h, and the cell lysates were analyzed by IB using an antibody against pSer. **C** HA–USP27X WT or HA–USP27X-S135A was co-transfected with or without Flag-GSK3β into HEK-293T cells. Cell lysates were immunoprecipitated using an anti-HA antibody and then analyzed by IB using a specific antibody against pSer. **D** Triple immunofluorescence (IF) staining of USP27X(green), GSK3β(red), and nuclei (DAPI, blue) was performed in MCF7 and MDA-MB-231 cells. Scale bar, 10 μm. **E** Cell lysates from MCF7 and MDA-MB-231 were analyzed by IP using antibodies against USP27X and GSK3β, then subjected to IB analysis. IgG was used as the isotype control. **F** BT549 cells were transfected with control vector or GSK3β for 36 h and then treated with 20 µM MG132 for 6 h. Cell lysates were immunoprecipitated using an antibody against USP27X and then analyzed by IB. **G**, **H** MDA-MB-231 (**G**) and MCF7 cells (**H**) were transfected with control siRNA or GSK3β siRNA for 36 h and then treated with 20 µM MG132 for 6 h. Cell lysates were immunoprecipitated using an antibody against USP27X and then analyzed by IB. **I** HEK-293T cells were transfected with Myc–CBX2, Flag–GSK3β and HA–USP27X-WT or HA–USP27X-S135A. Cell lysates were immunoprecipitated using an anti-Myc antibody and then analyzed by IB using an anti-HA antibody. **J** HEK-293T cells were transfected with His–Ub, Myc-CBX2, Flag–GSK3β, and HA–USP27X WT or HA–USP27X S135A. Cell lysates were immunoprecipitated using an anti-Myc antibody and then analyzed by IB using anti-Myc and anti-His antibodies. **K** BT549 cells were transfected with His–Ub, Myc-CBX2, HA–USP27X WT, or GSK3β siRNA. Cell lysates were immunoprecipitated using an anti-Myc antibody and then analyzed by IB using anti-Myc and anti-His antibodies. **L** BT549 cells transfected with USP27X WT, S135A, or S135Q mutants were treated with 100 μg/ml CHX, harvested at the indicated times, and treated with IB with anti-CBX2 and USP27X antibodies. Quantification of CBX2 levels relative to Vinculin is shown. **M** Relative CBX2 level of USP27X WT, S135A or S135Q mutants in BT549. For all panels, data are representative results of three independent experiments. Results were shown as mean ± SD; One-way ANOVA test, ***P < 0.001.

Our findings suggest that USP27X promotes BC progression through CBX2. However, the molecular mechanisms regulating USP27X in BC remain unclear, and strategies for directly targeting USP27X are yet to be established. Phosphorylation events can either generate or disrupt binding sites for DUBs, thereby influencing the stability and degradation of key proteins involved in cellular processes. Additionally, phosphorylation can regulate the deubiquitination activity of specific DUBs by modulating their catalytic activity, substrate specificity, or subcellular localization through phosphorylation of themselves or their interacting partners. The modulation of catalytic activity, substrate specificity, or subcellular localization of deubiquitinating enzymes (DUBs) can be achieved through their phosphorylation or that of their interacting partners. This modulation has a consequential impact on the deubiquitination process and downstream targets that are implicated in tumorigenesis [53]. We discovered in the PhosphoSitePlus PTM database that USP27X contains two potential GSK3β phosphorylation sites within its amino acid sequence (Fig. 5A). The administration of LiCI, a GSK3 inhibitor, led to a reduction in the levels of CBX2 phosphorylated serine in MDA-MB-231 and MCF7 cells (Fig. 5B). The phosphorylation of USP27X was solely observed in HEK-293T cells that were transfected with the wild-type USP27X, and not with the S135A mutant (Fig. 5C). Immunofluorescence staining confirmed the co-localization of GSK3β and USP27X primarily in the nucleus, with a lesser extent in the cytoplasm (Fig. 5D). Immunoprecipitation studies demonstrated that endogenous USP27X intercalates with GSK3β (Fig. 5E). Additionally, our findings suggest that Ser135 may be phosphorylated by GSK3β as it binds to the USP structural domain of USP27X (Supplementary Fig. 4A, B). Collectively, these results indicate that USP27X functions as a substrate for GSK3β. The upregulation of GSK3β in BT549 cells intensified the association between endogenous USP27X and CBX2 (Fig. 5F), whereas the downregulation of GSK3β in MDA-MB-231 and MCF7 cells weakened the interaction between endogenous USP27X and CBX2 (Fig. 5G, H). Furthermore, mutation of S135A on USP27X significantly reduced its binding affinity to CBX2 (Fig. 5I). The deubiquitination of CBX2 by USP27X S135A was found to be impaired in the presence of GSK3β (Fig. 5J), and a similar attenuation of CBX2 deubiquitination was observed upon knockdown of GSK3β (Fig. 5K). As anticipated, the capacity of USP27X mimetic phosphorylation-inactivated phenotype (USP27X S135A) to sustain CBX2 stability was significantly diminished compared to that of USP27X, while the ability of USP27X mimetic phosphorylation-activated phenotype (USP27X S135Q) to maintain CBX2 stability was considerably augmented (Fig. 5L, M). These findings suggest that GSK3β-mediated phosphorylation of USP27X is indispensable for its binding and deubiquitination with CBX2.

### The expression of USP27X in BC tissues is positively correlated with the levels of CBX2

**Fig. 6:**
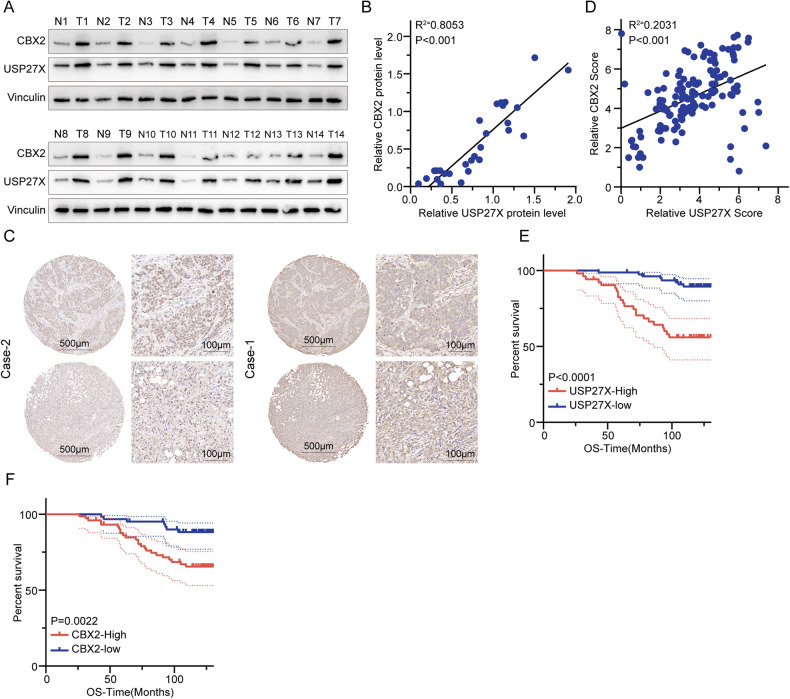
The expression of USP27X in BC tissues is positively correlated with the levels of CBX2. **A** IB detection of USP27X and CBX2 in BC specimens. **B** Correlation analysis of USP27X and CBX2 in BC tissues. Statistical analyses were performed with the *χ*^2^ test. The Pearson *r* indicates correlation coefficient. **C** Representative images of immunohistochemical staining of USP27X and CBX2 on tissue microarray of BC specimens (*n* = 132). Scale bar, 100 μm, 500 μm. **D** Relative CBX2 score = CBX2 protein expression/ Internal reference protein expression (Vinculin). Relative USP27X score = USP27X protein expression/ Internal reference protein expression (Vinculin). Data of IHC were analyzed using Pearson correlation by GraphPad Prism (8.3). **E**, **F** Kaplan–Meier curves showing overall survival of BC patients divided based on USP27X (**E**) and CBX2 expression (**F**) by GraphPad Prism (8.3). For all panels, data are representative results of three independent experiments.

To evaluate the clinical significance of the USP27X-CBX2 axis and its relevance in BC, we investigated the correlation between USP27X and CBX2 protein expression in human BC specimens. As depicted in Fig. 6A, B, a positive association was observed between USP27X and CBX2 protein levels in BC specimens (*P* < 0.0001, Pearson *r* = 0.9726). Subsequently, immunohistochemical staining for both USP27X and CBX2 was conducted on serial sections of BC tissue microarrays (*n* = 132). Representative images of USP27X and CBX2 staining are presented in Fig. 6C, indicating a significant positive correlation between the USP27X and CBX2 (*P* < 0.0001, Pearson *r* = 0.4372) (Fig. 6D). These findings suggest that upregulation of USP27X expression is associated with increased CBX2 expression in BC. Furthermore, Kaplan-Meier survival analysis was employed to evaluate the relationship between USP27X and CBX2 protein levels and clinical outcomes among BC patients. Clinical data demonstrated a significant correlation between elevated expression of either USP27X or CBX2 and reduced overall survival (Fig. 6E, F).

## Discussion

Chromobox protein homolog 2 (CBX2) has been identified as a multifaceted player in the progression of aggressive cancers [35]. The proteasome-dependent pathway is known to play a crucial role in regulating CBX2, but the regulatory roles and mechanisms of deubiquitinating enzymes that target CBX2 have remained elusive. The interaction between phosphorylation and deubiquitination, two pivotal post-translational modifications, exerts a notable influence on tumorigenesis and the advancement of cancer [54]. Phosphorylation denotes the attachment of phosphate groups to proteins, whereas deubiquitination involves the elimination of ubiquitin molecules from ubiquitinated proteins [55]. The interplay between these modifications influences protein stability, localization, and activity, ultimately impacting key signaling pathways involved in cancer. Phosphorylation can modulate the recognition and binding of deubiquitinases (DUBs) to ubiquitinated proteins, thereby influencing the process of deubiquitination [56]. In this study, we utilized mass spectrometry to identify ubiquitin-specific peptidase 27X (USP27X) as a deubiquitinating enzyme responsible for regulating CBX2. We have demonstrated that the overexpression of USP27X results in an increase in CBX2 levels through deubiquitination, while the deficiency of USP27X promotes CBX2 degradation and restrains tumorigenesis. Additionally, we have discovered a direct interaction between GSK3β and USP27X, where GSK3β phosphorylates USP27X to enhance its affinity for CBX2 and further stabilize CBX2 protein levels. Clinical analysis has revealed that the co-expression of elevated levels of USP27X and CBX2 in BC tissues is indicative of a poor prognosis. These findings collectively suggest that USP27X serves as a critical regulator of CBX2, and the GSK3β-USP27X-CBX2 axis plays a pivotal role in driving malignant progression in BC.

The discovery of USP27X as a deubiquitinating enzyme for CBX2 sheds light on the regulatory mechanisms controlling its stability, providing potential therapeutic targets for aggressive cancers. Our research emphasizes the significance of proteasome-dependent pathways in regulating CBX2 levels, supporting evidence that their dysfunction contributes to tumorigenesis. Through an investigation into the regulatory roles of deubiquitinating enzymes, we have discovered that USP27X is involved in CBX2 deubiquitination, shedding light on a previously unknown aspect of CBX2 regulation. One of the key findings in our study is that overexpression of USP27X significantly enhances the level of CBX2 through deubiquitination. This implies that USP27X functions as a positive regulator of CBX2, contributing to its stabilization and potentially promoting tumorigenesis. Conversely, the deficiency of USP27X results in CBX2 degradation, indicating a tumor-suppressive role for USP27X in restraining cancer progression. These findings emphasize the intricate interplay between deubiquitinating enzymes and the proteasome in regulating CBX2 levels and ultimately impacting tumorigenesis. Our study also elucidates the involvement of GSK3β in modulating the GSK3β-USP27X-CBX2 axis, revealing that GSK3β can directly interact with and phosphorylate USP27X, thereby enhancing its binding affinity for CBX2. This phosphorylation event enhances the stability of USP27X-CBX2 interaction, thereby promoting CBX2 protein accumulation. This novel regulatory mechanism expands our understanding of the intricate interplay between signaling pathways and post-translational modifications in cancer progression. Clinical analysis of BC tissues indicates that high co-expression levels of USP27X and CBX2 are associated with poor prognosis. This observation highlights the clinical significance of the GSK3β-USP27X-CBX2 axis as a promising prognostic marker in BC. The association between increased expression of these proteins and unfavorable patient outcomes emphasizes the necessity to consider USP27X and CBX2 as potential targets for BC therapy.

While our study offers valuable insights into the regulatory roles of USP27X and GSK3β in maintaining CBX2 stability, it is important to acknowledge certain limitations and areas for further investigation. Specifically, while we have identified USP27X as a deubiquitinating enzyme for CBX2 through mass spectrometry analysis, a more comprehensive understanding of the specific ubiquitination sites on CBX2 targeted by USP27X would enhance our knowledge of the underlying regulatory mechanisms. Additionally, exploring the potential interplay between USP27X and other deubiquitinating enzymes that may also modulate CBX2 levels would facilitate a comprehensive understanding of CBX2 regulation. Moreover, while our study highlights the role of GSK3β in phosphorylating USP27X and enhancing its affinity for CBX2, the downstream signaling events triggered by this interaction remain largely unexplored. Understanding the signaling cascades and downstream effectors influenced by the GSK3β-USP27X-CBX2 axis could provide further insights into the molecular mechanisms driving malignant progression in BC. Additionally, our study is limited to BC, and further investigations are necessary to determine the role of USP27X and the GSK3β-USP27X-CBX2 axis in other types of cancer. Different types of cancer may present unique molecular characteristics and signaling pathways, thus the impact of USP27X and CBX2 could vary. Examining the expression and functional significance of these genes across a wider range of cancer types would enhance the translational potential of our findings. Moreover, although our clinical analysis has revealed a correlation between elevated levels of USP27X and CBX2 protein levels and unfavorable prognosis in BC patients, it is imperative to conduct larger-scale studies with comprehensive patient cohorts to validate these findings. Additionally, conducting functional studies to investigate the impact of targeting USP27X and CBX2 in preclinical models and assessing their therapeutic potential would be beneficial for translating these findings into clinical applications.

In summary, our study provides significant insights into the regulatory roles of USP27X and GSK3β in maintaining the stability of CBX2, underscoring their importance in driving malignant progression in BC. However, further investigations are necessary to identify specific ubiquitination sites on CBX2, explore potential interactions with other deubiquitinating enzymes, investigate downstream signaling events, and extend the scope of this research to other types of cancer. Furthermore, to confirm the clinical significance and therapeutic potential of targeting USP27X and CBX2 in cancer treatment, more extensive clinical studies and functional preclinical studies are required. Overcoming these limitations will enhance our comprehension of the interplay between post-translational modifications and lay a solid foundation for developing targeted therapies against aggressive cancers.

## Methods

### Study approval

The Animal Research Committee of Inner Mongolia Medical University granted approval for the animal experiments, and strict adherence to the guidelines for animal care at Inner Mongolia University was observed during housing and handling of the animals. Data on patients and their tumors were collected through medical records and pathological reports. The experimental procedures were approved by the ethics committee of Inner Mongolia Medical University, and written informed consent was obtained from all participants.

### Plasmids

The HA-USP27X and HA-USP27X C87A constructs were generated by cloning human USP27X cDNA into the pcDNA3.1-HA vector, while the Myc-CBX2 construct was produced through the insertion of human CBX2 cDNA into the pcDNA3.1-Myc vector. Additionally, both His-Ub and Flag-GSK3β constructs were created by incorporating human Ub and GSK3β cDNA into their respective vectors, pcDNA3.1-His and pcDNA3.1-Flag. The lentiviral vector expressing USP27X was generated by cloning USP27X cDNA into the pLVX-Puro-HA vector. To construct truncated mutants of USP27X and CBX2, the corresponding human cDNA were cloned into pcDNA3.1 using HA-USP27X and Myc-CBX2 as templates. The GST-USP27X and GST-USP27X C87A constructs were generated by cloning the corresponding human cDNA into pGEX-1λT, a modified version of pGEX-4T1. Site-directed mutagenesis was employed to introduce point mutations, using the respective plasmids as templates.

### Antibodies and other reagents

The antibodies targeting the proteins/epitopes Myc (2276), HA (3724), His (12698), Flag (14793), Ub (3936), Vinculin (13901), GSK3β (12456), and CBX2 (25069) were procured from Cell Signaling Technology. The USP27X antibody (AP16881a) was procured from Abcepta, while the USP27X for IF (NBP2-57733) was obtained from NOVUS. The phosphoserine antibody (ER60006) and CBX2 antibody (EM1706-28) were acquired from HUABIO. Additionally, the Anti-Myc Magnetic Beads (HY-K0206A), Anti-HA Magnetic Beads (HY-K0201A), Anti-Flag Magnetic Beads (HY-K0207A), Z-Leu-Leu-Leu-al (MG132, HY-13259), and Cycloheximide (HY-12320) were purchased from MedChemExpress.

### Cell culture

The cells utilized in this study were procured from Procell, with the exception of those specifically noted. HEK-293T cells were maintained in DMEM, while MDA-MB-231 cells were cultured in Leibovitz’s L-15. MCF7 cells were cultured in MEM, and BT-549 cells were cultured in RPMI 1640.

### Transient and stable RNAi knockdown

To achieve transient knockdown, the cells were subjected to siRNA transfection using Lipofectamine 3000 (Invitrogen Life Technologies) in accordance with the manufacturer’s guidelines. The siRNA sequences employed were as follows: negative control, 5′-TACAAACGCTCTCATCGACAAG-3′; GSK-3β 1, 5′-CAGCTGGTGGAGATCATCA-3′; GSK-3β 2, 5′-CAGGACAAGAGGTTCAAGA-3′; GSK-3β 3, 5′-AGCCCAATGTCTCCTACAT-3′. To achieve stable knockdown, lentiviral particles expressing shRNAs were utilized to infect cells, followed by selection in the presence of puromycin. The shRNA sequences employed were as follows: shUSP27X 1, 5′-ACCTCCAGCTTTACGATCG-3′; shUSP27X 2, 5′-GACAGGCACCGATGTGAGA-3′; shUSP27X 3, 5′-GAGGCGCAAGATCACTACA-3′; shCBX2 1, 5′-TGCGTTGTTGCTGAGTTTG-3′; shCBX2 2, 5′-TGACAGTGACTTAGATGCT-3′; shCBX2 3, 5′-AGCATGTTCCATTTCTAAA-3′, as previously described.

### GST pull-down assay

The bacterial expression plasmid pGEX-4T-1 was utilized to express both GST-USP27X-WT and GST-USP27X-C87A in Escherichia coli strain BL21. The resulting proteins were purified using Sigma-Aldrich GST beads according to the manufacturer’s instructions. For the GST pull-down assay, bacterial-expressed GST, GST-USP27X, or GST-USP27X-C87A bound to glutathione-Sepharose 4B beads (GE Healthcare) were incubated with His-Myc-CBX2 expressed in HEK-293T cells for 2 h at 4 °C.

### Immunoblotting and co-IP assay

To conduct immunoblotting (IB), cells were lysed in a lysis buffer containing 50 mM Tris-Cl at pH 7.4, 150 mM NaCl, 1% Triton X-100, and 1 mM DTT supplemented with a protease and phosphatase inhibitor cocktail (MCE). For co-IP, designated antibodies were utilized to immunoprecipitate the cell lysates. The resulting immunoprecipitates underwent four washes with lysis buffer before being analyzed via SDS-PAGE and IB.

### RNA isolation and quantitative real-time PCR

The RNeasy Mini Kit (QIAGEN) was employed for the extraction of Total RNA, followed by reverse transcription using an iScript cDNA Synthesis Kit (Bio-Rad). The expression levels of USP27X and CBX2 were determined using the 2–ΔΔCt method and normalized to the housekeeping gene GAPDH. Primer sequences for USP27X and CBX2 are provided in Supplementary Table 1.

### Immunohistochemistry

The human BC tissue microarray was deparaffinized and rehydrated using a descending alcohol series for immunohistochemical staining. Subsequently, antigen retrieval was performed with sodium citrate buffer. The tumor sections were blocked with 1% BSA containing 0.25% Triton X-100 and 3% H_2_O_2_ in PBS for one hour at room temperature, followed by overnight incubation with the specified primary antibodies at 4 °C. Subsequently, HRP conjugates were administered and diaminobenzidine detection was employed.

### In vivo and in vitro deubiquitylation of CBX2

For in vivo experiments, after transfection with the indicated plasmids, cells were treated with 20 μM MG132 for 8 h and detected for CBX2 ubiquitination. Subsequently, cells were harvested and lysed in RIPA buffer. The lysate was then incubated with anti-CBX2 antibody for 3 h, followed by protein A /G agarose beads for an additional 8 h at a temperature of 4 °C. The plates were then boiled in SDS-PAGE loading buffer for 10 min followed by IB treatment with anti-ubiquitin antibody. HEK-293T cells were transfected with Myc-CBX2 and His-ubiquitin and treated with 20 μM MG132 for 8 h to prepare ubiquitylated CBX2 as a substrate for in vitro deubiquitination experiments. Ubiquitinated CBX2 was isolated from cell extracts using anti-Myc magnetic beads and subsequently incubated with purified HA-USP27X-WT or HA-USP27X-C87A proteins for 2 h at 37 °C in deubiquitination buffer. The resulting responses were then analyzed by IB analysis.

### Protein half-life assay

The cells that were transfected with the plasmids specified were subjected to treatment with CHX (50 μg/ml), an inhibitor of protein synthesis, for the durations indicated prior to being collected.

### Proximity Ligation Assay

To commence proximity ligation, cells were initially cultivated on collagen-coated Lab-Tek II chamber slides for a minimum of 16 h. Following this, the resultant cells underwent two washes with PBS and were subsequently fixed with 3.7% formaldehyde in PBS for 15 min at room temperature. The slides were then subjected to a wash with TBS (25 mM Tris, 100 mM NaCl, pH 7.4) and treated with 50 mM NH4Cl in TBS for 10 min. After another wash with TBS, the cells were rendered permeable with 0.1% Triton X-100 in TBS for 15 min and then washed again with TBST. Subsequently, the slides were treated with 0.5% Roche Applied Science milk powder in TBST for 2 h at 37 °C in a humidified chamber to prevent any further movement. The slides were then incubated overnight at 4 °C with a suitable combination of antibodies and washed with TBST. The proximity ligation was carried out using the Rabbit PLUS and Mouse MINUS Duolink in situ PLA kits (Sigma-Aldrich) as per the manufacturer’s guidelines. The slides were subjected to dehydration and air-drying, and subsequently embedded in a Citifluor mounting medium containing DAPI (Sigma-Aldrich). The analysis of PLA in fresh-frozen tumor tissue involved fixation of cryosections with 3.7% formaldehyde in PBS for 15 min, followed by execution of in situ PLA reactions using the same protocol as for cultured cells.

### Immunofluorescence

To perform immunofluorescence staining, the cells were initially fixed using a 4% formaldehyde solution, followed by permeabilization using 0.25% Triton X-100. Subsequently, the cells were blocked with 1% BSA for a duration of 1 h at room temperature. The cells were then probed with the primary antibodies as indicated.

### Colony formation and cell migration assays

For colony formation assays, single-cell suspensions of cells overexpressing specific genes were diluted, cultured in 6-well plates at a density of 1000 cells per well, and incubated at 37 °C and 5% CO_2_ for 2 weeks. Colonies were then stained with 0.04% crystal violet and quantified. Cell migration assays were performed using 24-well transwell plates containing 8 μm polyethylene terephthalate filter membrane (Corning) and migration was performed for 24 h in a humidified chamber. After the incubation period, filters were extracted and treated with 4% formaldehyde solution for 15 min. Cells located in the lower filter membrane were subsequently stained with 0.1% crystal violet solution for 20 min and counted.

### In vivo tumorigenesis and metastasis assay

The Institutional Animal Care and Use Committee (Inner Mongolia Medical University Medical Ethics Committee), affiliated with Inner Mongolia Medical University, approved all animal experiments. Specifically, 4-week-old female BALB/c nude mice (*n* = 5) (spfbiotech, Beijing) were subcutaneously implanted with MDA-MB-231 and BT549 cells (1 × 10^6^ cells). The tumor’s short and long diameters were measured using a vernier caliper, and the tumor volume was assessed every 3 days. The tumor volume was calculated using the formula width^2^ × length × 0.5 (mm^3^). For the purpose of investigating lung metastasis, 1×10^6^ MDA-MB-231 and BT549 cells were injected into the mammary fat pad of female NOD-SCID mice (5-6 weeks old, *n* = 6) obtained from Spfbiotech Company. Once the primary tumor reached a size of 400 mm^3^, it was removed. After an 8-week interval, the mice were euthanized and the number of pulmonary metastatic nodules was counted and quantified.

### Mass Spectrometry

PTM BIO provides resources and services for mass spectrometry proteomics. Methods procedures roughly involved the preparation of protein samples and the digestion of proteins present in the gel bands by overnight incubation with trypsin. Additional peptides will be extracted from the gel and then shaken for 10 min. The supernatant was then dried, after which it was reconstituted and eluted with 0.1% formic acid for 5 min before final mass spectrometry (Supplementary Table 2).

### Statistical analysis

The data in all graphs is presented using the mean ± standard deviation of biological triplicates. Statistical significance is determined using GraphPad Prism 8.3 software, which employs a 2-tailed Student’s t test or 1-way ANOVA with Dunnett’s post-test for multiple comparisons. The statistical significance of Kaplan-Meier survival curves is determined through a log-rank (Mantel-Cox) analysis. The *P* value of <0.05 is considered significant for all statistical tests.

### Supplementary information

Supplement Figure 1

Supplement Figure 2

Supplement Figure 3

Supplement Figure 4

Supplementary Figure Legends

Supplementary Table 1

Supplementary Table 2

Original Data File

A reproducibility checklist

## Data Availability

All datasets generated and analyzed during this study are included in this published article and its Supplementary Information files. All data generated or analyzed during this study are available from the corresponding author on reasonable request.

## References

[1] Nolan E, Lindeman GJ, Visvader JE (2023). Deciphering breast cancer: from biology to the clinic. Cell.

[2] Penault-Llorca F, Viale G (2012). Pathological and molecular diagnosis of triple-negative breast cancer: a clinical perspective. Ann Oncol.

[3] Boyle P (2012). Triple-negative breast cancer: epidemiological considerations and recommendations. Ann Oncol.

[4] Fernandez SV, Bingham C, Fittipaldi P, Austin L, Palazzo J, Palmer G (2014). TP53 mutations detected in circulating tumor cells present in the blood of metastatic triple negative breast cancer patients. Breast Cancer Res.

[5] Ferraro DA, Gaborit N, Maron R, Cohen-Dvashi H, Porat Z, Pareja F (2013). Inhibition of triple-negative breast cancer models by combinations of antibodies to EGFR. Proc Natl Acad Sci USA.

[6] Shi H, Wang M, Huang J, Ouyang Q, Guo J, Wang Y (2023). Abstract 4021: CTS2016, a novel AXL/FLT3 inhibitor for targeting AML/MDS and solid tumors. Cancer Res.

[7] Xu Z, Goel HL, Burkart C, Burman L, Chong YE, Barber AG (2023). Inhibition of VEGF binding to neuropilin-2 enhances chemosensitivity and inhibits metastasis in triple-negative breast cancer. Sci Transl Med.

[8] Abu Alragheb BO, Abushukair H, Al-Husseini M, Bawadi R, Ahram M (2023). Abstract 3772: the association of androgen receptor with microRNA expression in triple-negative breast cancer. Cancer Res.

[9] Parnigoni A, Caon I, Teo WX, Hua SH, Moretto P, Bartolini B (2022). The natural antisense transcript HAS2-AS1 regulates breast cancer cells aggressiveness independently from hyaluronan metabolism. Matrix Biol.

[10] Sun T, Liu Z, Yang Q (2020). The role of ubiquitination and deubiquitination in cancer metabolism. Mol Cancer.

[11] Mukhopadhyay D, Riezman H (2007). Proteasome-independent functions of ubiquitin in endocytosis and signaling. Science.

[12] Metzger MB, Pruneda JN, Klevit RE, Weissman AM (2014). RING-type E3 ligases: master manipulators of E2 ubiquitin-conjugating enzymes and ubiquitination. Biochim Biophys Acta.

[13] Deng L, Meng T, Chen L, Wei W, Wang P (2020). The role of ubiquitination in tumorigenesis and targeted drug discovery. Signal Transduct Target Ther.

[14] Abdul Rehman SA, Kristariyanto YA, Choi SY, Nkosi PJ, Weidlich S, Labib K (2016). MINDY-1 is a member of an evolutionarily conserved and structurally distinct new family of deubiquitinating enzymes. Mol Cell.

[15] Yu L, Liu P (2021). Cytosolic DNA sensing by cGAS: regulation, function, and human diseases. Signal Transduct Target Ther.

[16] Koppens M, van Lohuizen M (2016). Context-dependent actions of Polycomb repressors in cancer. Oncogene.

[17] Dong L, Yu L, Bai C, Liu L, Long H, Shi L (2018). USP27-mediated Cyclin E stabilization drives cell cycle progression and hepatocellular tumorigenesis. Oncogene.

[18] Kosinsky RL, Zerche M, Saul D, Wang X, Wohn L, Wegwitz F (2020). USP22 exerts tumor-suppressive functions in colorectal cancer by decreasing mTOR activity. Cell Death Differ.

[19] Seo SU, Woo SM, Kim MW, Lee HS, Kim SH, Kang SC (2020). Cathepsin K inhibition-induced mitochondrial ROS enhances sensitivity of cancer cells to anti-cancer drugs through USP27x-mediated Bim protein stabilization. Redox Biol.

[20] Siebzehnrubl FA, Silver DJ, Tugertimur B, Deleyrolle LP, Siebzehnrubl D, Sarkisian MR (2013). The ZEB1 pathway links glioblastoma initiation, invasion and chemoresistance. EMBO Mol Med.

[21] Lambies G, Miceli M, Martínez-Guillamon C, Olivera-Salguero R, Peña R, Frías CP (2019). TGFβ-activated USP27X deubiquitinase regulates cell migration and chemoresistance via stabilization of snail1. Cancer Res.

[22] Clermont PL, Sun L, Crea F, Thu KL, Zhang A, Parolia A (2014). Genotranscriptomic meta-analysis of the Polycomb gene CBX2 in human cancers: initial evidence of an oncogenic role. Br J Cancer.

[23] Wheeler LJ, Watson ZL, Qamar L, Yamamoto TM, Post MD, Berning AA (2018). CBX2 identified as driver of anoikis escape and dissemination in high grade serous ovarian cancer. Oncogenesis.

[24] Liang YK, Lin HY, Chen CF, Zeng D (2017). Prognostic values of distinct CBX family members in breast cancer. Oncotarget.

[25] Piqué DG, Montagna C, Greally JM, Mar JC (2019). A novel approach to modelling transcriptional heterogeneity identifies the oncogene candidate CBX2 in invasive breast carcinoma. Br J Cancer.

[26] Li X, Gou J, Li H, Yang X (2020). Bioinformatic analysis of the expression and prognostic value of chromobox family proteins in human breast cancer. Sci Rep.

[27] Di Costanzo A, Del Gaudio N, Conte L, Dell’Aversana C, Vermeulen M, de Thé H (2018). The HDAC inhibitor SAHA regulates CBX2 stability via a SUMO-triggered ubiquitin-mediated pathway in leukemia. Oncogene.

[28] Mutoh H, Sakurai S, Satoh K, Tamada K, Kita H, Osawa H (2004). Development of gastric carcinoma from intestinal metaplasia in Cdx2-transgenic mice. Cancer Res.

[29] Lee NP, Poon RT, Shek FH, Ng IO, Luk JM (2010). Role of cadherin-17 in oncogenesis and potential therapeutic implications in hepatocellular carcinoma. Biochim Biophys Acta.

[30] Crea F, Hurt EM, Farrar WL (2010). Clinical significance of polycomb gene expression in brain tumors. Mol Cancer.

[31] Hu FF, Chen H, Duan Y, Lan B, Liu CJ, Hu H (2022). CBX2 and EZH2 cooperatively promote the growth and metastasis of lung adenocarcinoma. Mol Ther Nucleic Acids.

[32] Xu Z, Wu Y, Yang M, Wei H, Pu J (2023). CBX2-mediated suppression of SIAH2 triggers WNK1 accumulations to promote glycolysis in hepatocellular carcinoma. Exp Cell Res.

[33] Zhou H, Xiong Y, Liu Z, Hou S, Zhou T (2021). Expression and prognostic significance of CBX2 in colorectal cancer: database mining for CBX family members in malignancies and vitro analyses. Cancer Cell Int.

[34] Zheng S, Lv P, Su J, Miao K, Xu H, Li M (2019). Overexpression of CBX2 in breast cancer promotes tumor progression through the PI3K/AKT signaling pathway. Am J Transl Res.

[35] Iqbal MA, Siddiqui S, Ur Rehman A, Siddiqui FA, Singh P, Kumar B (2021). Multiomics integrative analysis reveals antagonistic roles of CBX2 and CBX7 in metabolic reprogramming of breast cancer. Mol Oncol.

[36] Del Gaudio N, Di Costanzo A, Liu NQ (2022). CBX2 shapes chromatin accessibility promoting AML via p38 MAPK signaling pathway[J]. Mol Cancer.

[37] Kessler BM, Edelmann MJ (2011). PTMs in conversation: activity and function of deubiquitinating enzymes regulated via post-translational modifications. Cell Biochem Biophys.

[38] de Poot SAH, Tian G, Finley D (2017). Meddling with fate: the proteasomal deubiquitinating enzymes. J Mol Biol.

[39] Seo SU, Woo SM, Kim MW, Lee EW, Min KJ, Kwon TK (2023). Phosphorylation of OTUB1 at Tyr 26 stabilizes the mTORC1 component, Raptor. Cell Death Differ.

[40] Walser F, Mulder MPC, Bragantini B, Burger S, Gubser T, Gatti M (2020). Ubiquitin phosphorylation at Thr12 modulates the DNA damage response. Mol Cell.

[41] Das T, Kim EE, Song EJ (2019). Phosphorylation of USP15 and USP4 regulates localization and spliceosomal deubiquitination. J Mol Biol.

[42] Alkalay I, Yaron A, Hatzubai A, Orian A, Ciechanover A, Ben-Neriah Y (1995). Stimulation-dependent I kappa B alpha phosphorylation marks the NF-kappa B inhibitor for degradation via the ubiquitin-proteasome pathway. Proc Natl Acad Sci USA.

[43] Nalepa G, Rolfe M, Harper JW (2006). Drug discovery in the ubiquitin-proteasome system. Nat Rev Drug Discov.

[44] Li HH, Du J, Fan YN, Zhang ML, Liu DP, Li L (2011). The ubiquitin ligase MuRF1 protects against cardiac ischemia/reperfusion injury by its proteasome-dependent degradation of phospho-c-Jun. Am J Pathol.

[45] Pozhidaeva A, Bezsonova I (2019). USP7: structure, substrate specificity, and inhibition. DNA Repair.

[46] Lange SM, Armstrong LA, Kulathu Y (2022). Deubiquitinases: from mechanisms to their inhibition by small molecules. Mol Cell.

[47] López-Otín C, Hunter T (2010). The regulatory crosstalk between kinases and proteases in cancer. Nat Rev Cancer.

[48] Ye Y, Blaser G, Horrocks MH, Ruedas-Rama MJ, Ibrahim S, Zhukov AA (2012). Ubiquitin chain conformation regulates recognition and activity of interacting proteins. Nature.

[49] Zhou H et al. SENP3 and USP7 regulate Polycomb-rixosome interactions and silencing functions[J]. Cell Rep. 2023,42:112339. 10.1016/j.celrep.2023.112339 PMC10777863 37014752

[50] Vamisetti GB, Saha A, Huang YJ, Vanjari R, Mann G, Gutbrod J (2022). Selective macrocyclic peptide modulators of Lys63-linked ubiquitin chains disrupt DNA damage repair. Nat Commun.

[51] Sun D, Cao X, Wang C (2019). Polycomb chromobox Cbx2 enhances antiviral innate immunity by promoting Jmjd3-mediated demethylation of H3K27 at the Ifnb promoter. Protein Cell.

[52] Chan HL, Morey L (2019). Emerging roles for polycomb-group proteins in stem cells and cancer. Trends Biochem Sci.

[53] Wang X, Xia S, Li H, Wang X, Li C, Chao Y (2020). The deubiquitinase USP10 regulates KLF4 stability and suppresses lung tumorigenesis. Cell Death Differ.

[54] Wu Z, Huang R, Yuan L (2019). Crosstalk of intracellular post-translational modifications in cancer. Arch Biochem Biophys.

[55] Ribet D, Cossart P (2010). Pathogen-mediated posttranslational modifications: a re-emerging field. Cell.

[56] Hunter T (2007). The age of crosstalk: phosphorylation, ubiquitination, and beyond. Mol Cell.

