# Supplementary figures and images for: Phosphorylation of USP27X by GSK3β maintains the stability and oncogenic functions of CBX2
Source: Cell Death Dis. 2023 Nov 29;14(11):782. doi: 10.1038/s41419-023-06304-y (PMC10687032; doi:10.1038/s41419-023-06304-y)

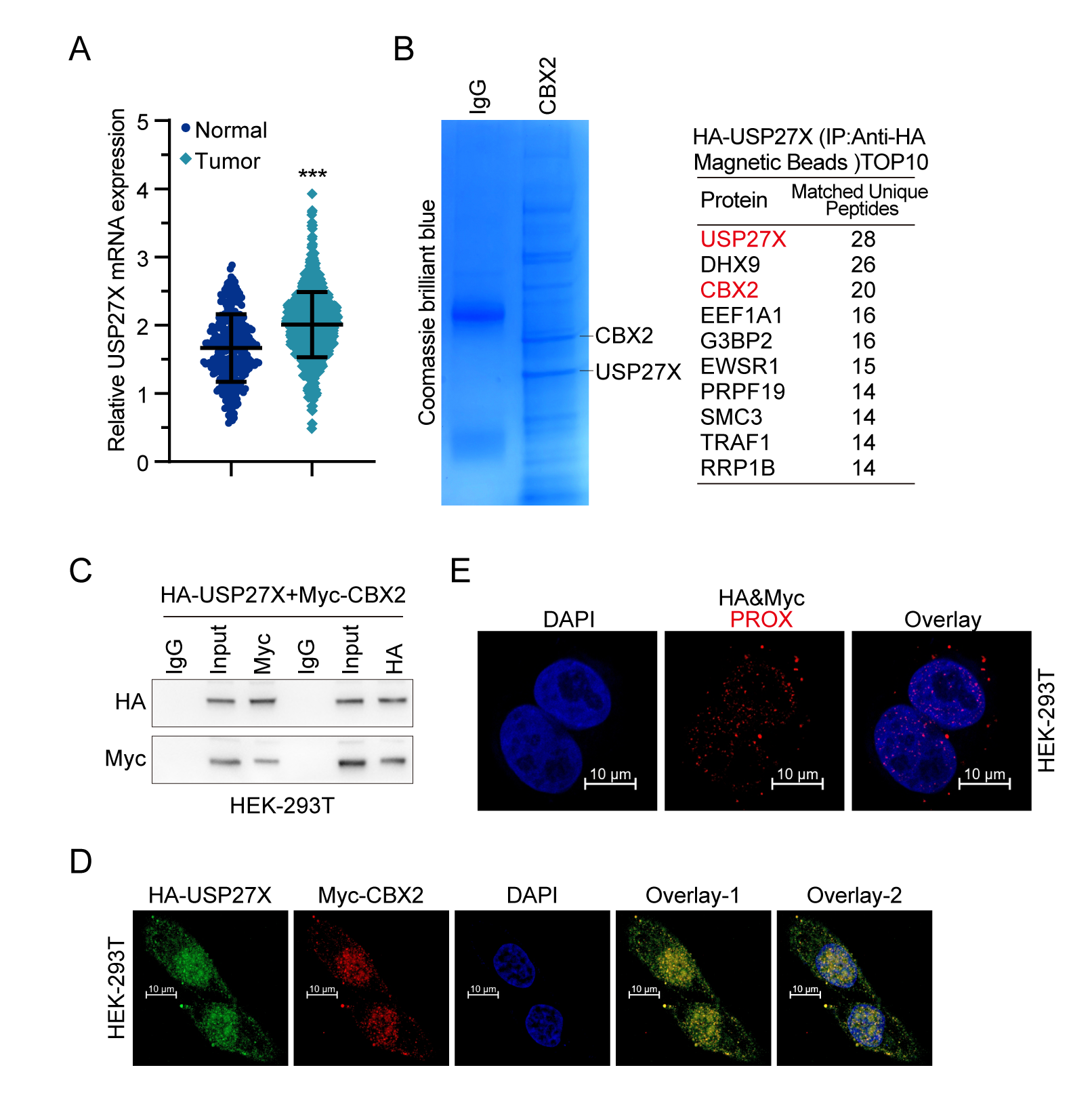

Supplement: Supplementary file 1 — Supplement Figure 1 [file 41419_2023_6304_MOESM1_ESM.tif]

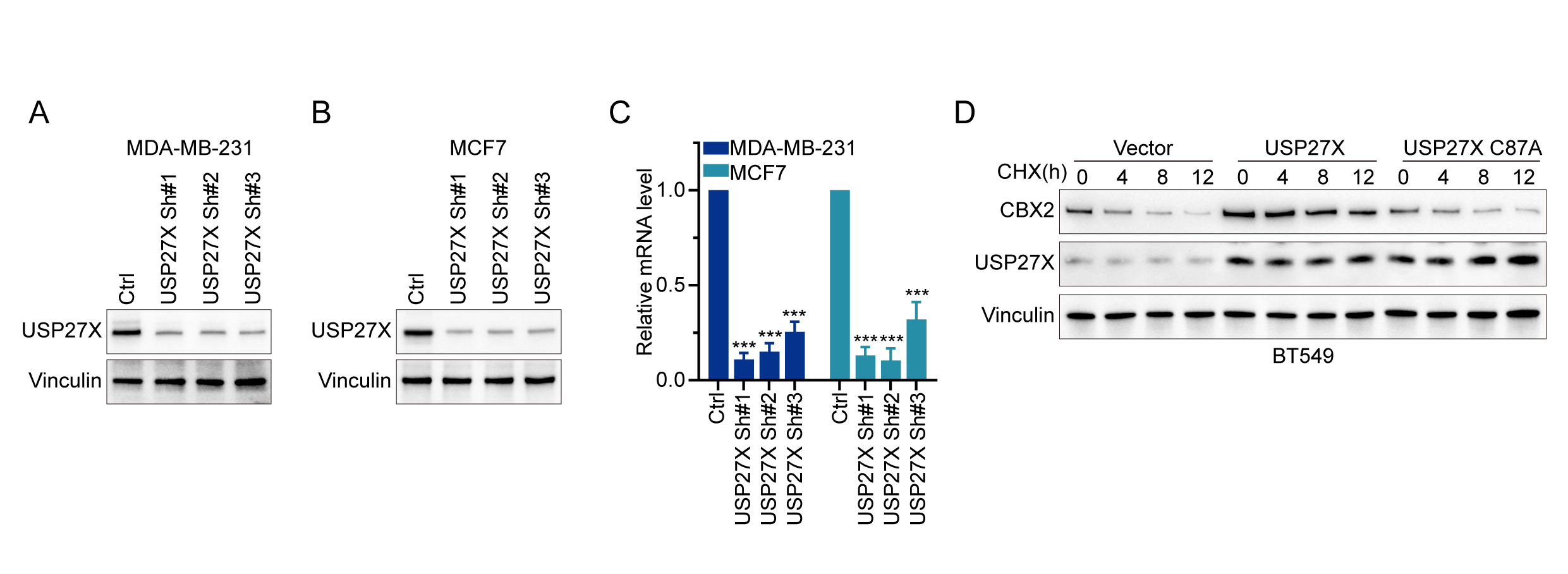

Supplement: Supplementary file 2 — Supplement Figure 2 [file 41419_2023_6304_MOESM2_ESM.tif]

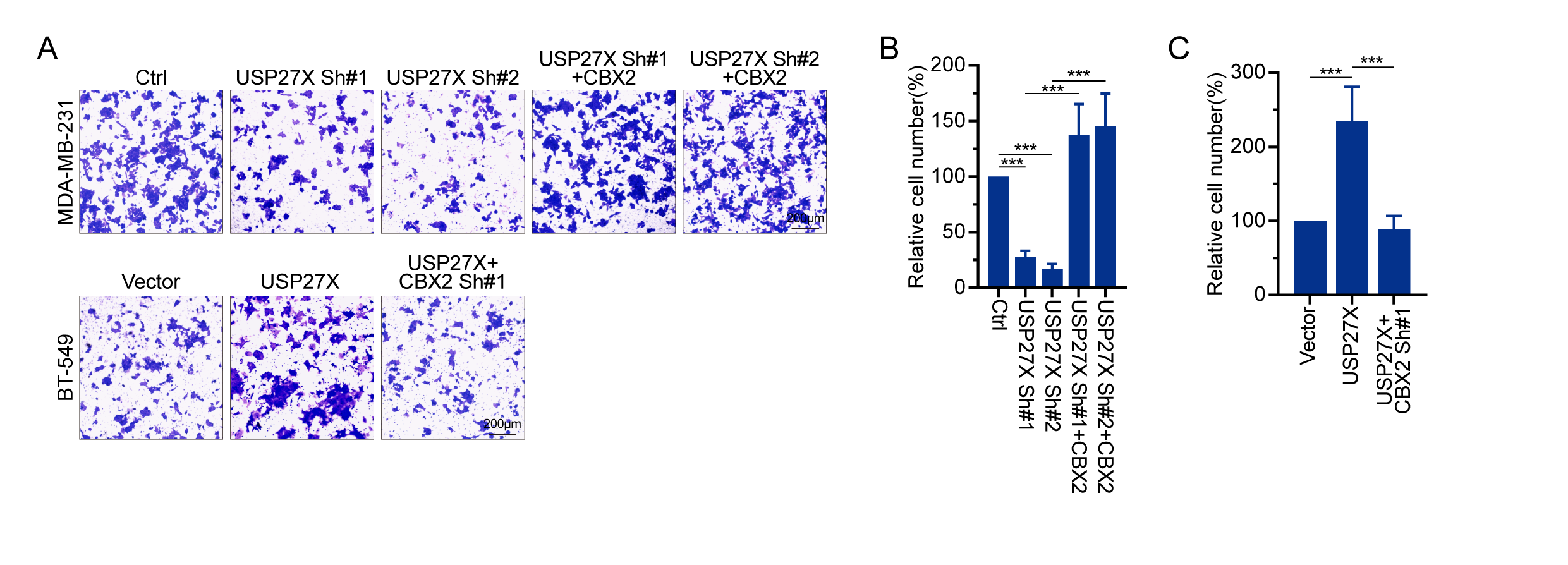

Supplement: Supplementary file 3 — Supplement Figure 3 [file 41419_2023_6304_MOESM3_ESM.tif]

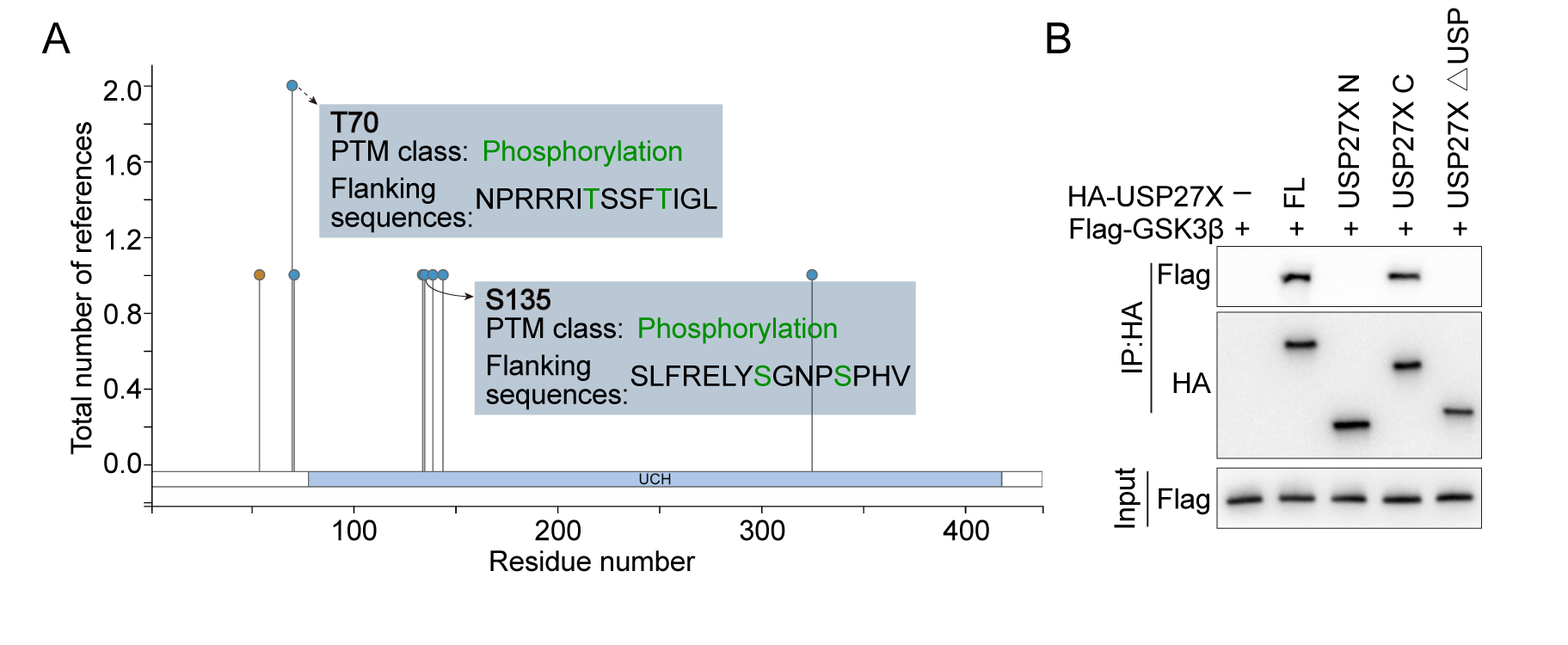

Supplement: Supplementary file 4 — Supplement Figure 4 [file 41419_2023_6304_MOESM4_ESM.tif]
