# Supplementary material for: Phosphorylation of USP27X by GSK3β maintains the stability and oncogenic functions of CBX2
Source: Cell Death Dis. 2023 Nov 29;14(11):782. doi: 10.1038/s41419-023-06304-y (PMC10687032; doi:10.1038/s41419-023-06304-y)
Supplement: Supplementary file 5 — Supplementary Figure Legends [file 41419_2023_6304_MOESM5_ESM.docx]

**Supplementary Figure Legends**

**Supplementary Fig. 1 USP27X interacts with CBX2.**

**(A)** Relative USP27X mRNA expression of normal and tumor tissues of TCGA. **(B)** The representative image of coomassie brilliant blue staining of USP27X in CBX2 precipitation. **(C)** HA-USP27X and Myc-CBX2 were co-transfected into HEK-293T cells. Cell lysates were immunoprecipitated with anti-HA or anti-Myc antibodies. **(D)** Triple immunofluorescence (IF) staining of exogenous HA-USP27X(green), Myc-CBX2(red), and nucleus (DAPI, blue) was performed in HEK-293T cells. Scale bar, 10 μm. **(E)** Representative images show the fusion of PLA with nuclear (DAPI) channels. In situ PLA between exogenous USP27X and CBX2. Scale bar, 10 μm. For all panels, data are representative results of three independent experiments. Results were shown as mean ± SD; One-way ANOVA test, ****P* < 0.001.

**Supplementary Fig. 2 USP27X enhances CBX2 protein stability.**

**(A-B)** MDA-MB-231 **(A)** and MCF7 **(B)** cells were transfected with 3 independent USP27X shRNAs. USP27X protein expression level was detected by IB analysis. **(C)** MDA-MB-231 **(A)** and MCF7 **(B)** cells were transfected with 3 independent USP27X shRNAs. USP27X mRNA level was detected by qRT-PCR analysis. **(D)** BT549 cells transfected with USP27X WT or C87A mutants were treated with 100 μg/ml CHX, harvested at the indicated times, and treated with IB with anti-CBX2 and USP27X antibodies. For all panels, data are representative results of three independent experiments. Data are represented as mean ± SD of 3 independent experiments. ****P* < 0.001, 1-way ANOVA with Dunnett’s post test (C).

**Supplementary Fig. 3 USP27X promotes the proliferation, invasion and metastasis of BC cells by up-regulating CBX2.**

**(A-C)** The indicated cells migration was examined by transwell assay. **(A)** In MDA-MB-231, two knockdown sequences of USP27X (USP27X Sh#1 and USP27X Sh#2) were constructed, and it was found that knockdown USP27X could significantly reduce the invasion ability of BC cells. USP27X combined overexpression of CBX2 can reverse this phenomenon to some extent. In BT549, overexpression of USP27X can increase tumor cell invasion, while overexpressed USP27X combined knockdown CBX2 can reverse this phenomenon. Scale bar: 200 μm. **(B)** Relative cell number of Supplementary Fig. 3A in MDA-MB-231. **(C)** Relative cell number of Supplementary Fig. 3A in BT549. Data are represented as mean ± SD of 3 independent experiments. ****P* < 0.001, 1-way ANOVA with Dunnett’s post test (B and C).

**Supplementary Fig. 4 GSK3β enhances the stability of CBX2 protein by phosphorylating USP27X.**

**(A)** Proteomics database (PhosphoSitePlus) analysis indicated that several amino acid residues of USP27X may be phosphorylated. **(B)** HEK-293T cells were cotransfected with Flag-GSK3β and HA-tagged FL USP27X or its deletion mutants and anti-Flag or HA-coupled magnetic beads for IP analysis of cell lysates followed by IB analysis with HA and Flag antibodies. For all panels, data are representative results of three independent experiments.
