# Supplementary material for: Phosphorylation of USP27X by GSK3β maintains the stability and oncogenic functions of CBX2
Source: Cell Death Dis. 2023 Nov 29;14(11):782. doi: 10.1038/s41419-023-06304-y (PMC10687032; doi:10.1038/s41419-023-06304-y)

Uncropped blots Related to Figure 1

A

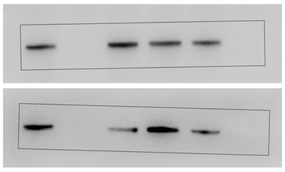

B

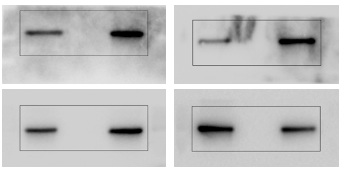

C

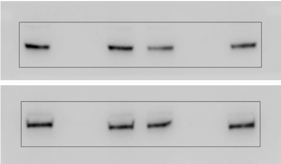

E

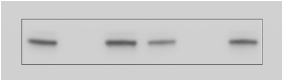

I

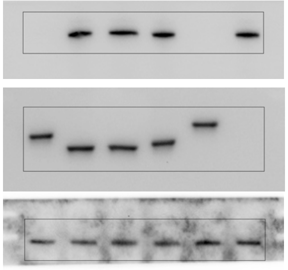

J

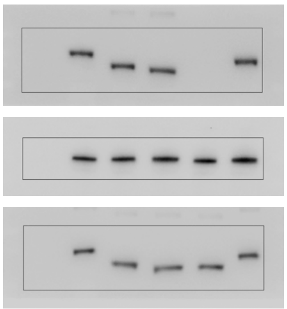

K

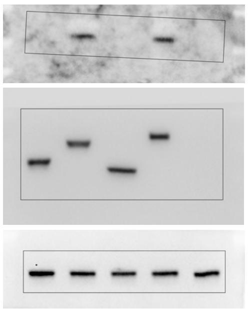

L

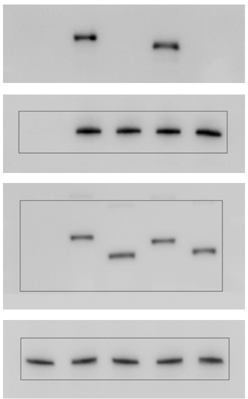

S1C

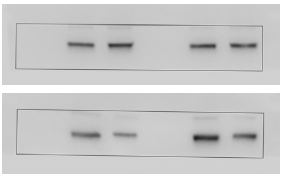

Uncropped blots Related to Figure 2

A

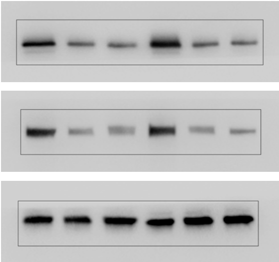

B

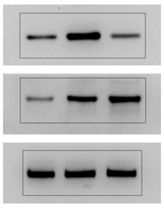

C

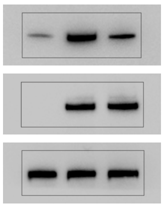

G

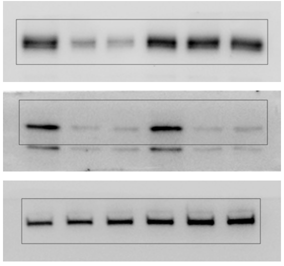

H

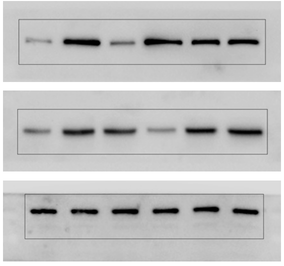

F

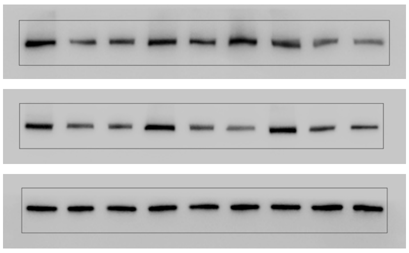

I

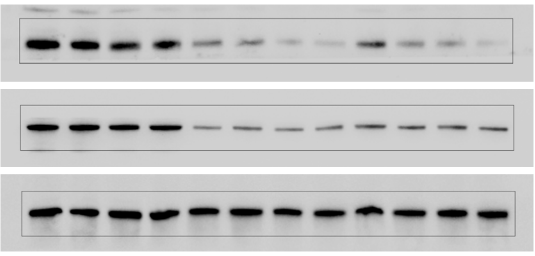

S2A

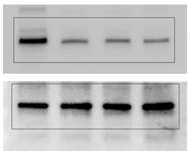

S2B

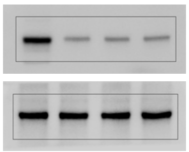

K

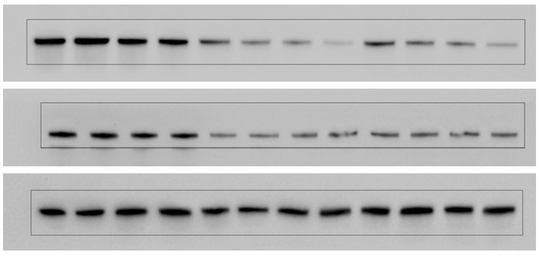

S2D

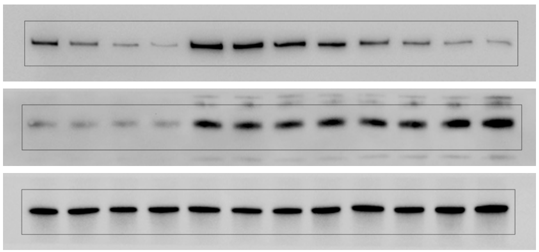

Uncropped blots Related to Figure 3

A

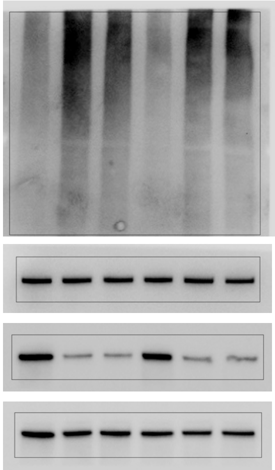

B

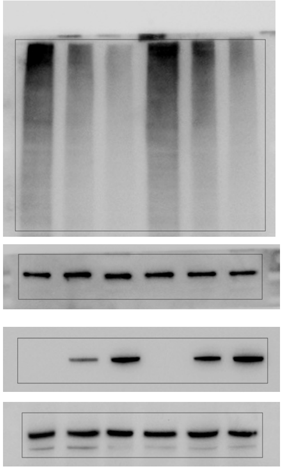

C

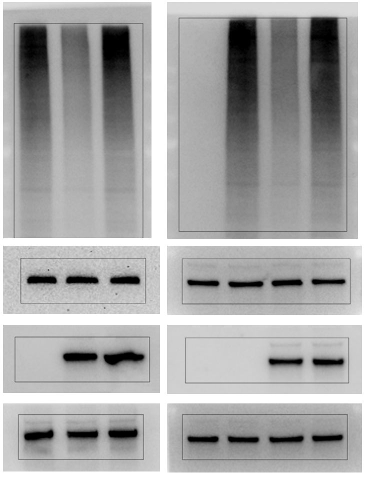

D

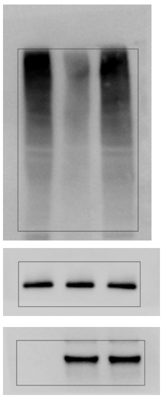

E

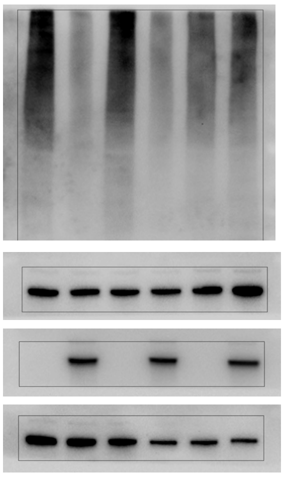

F

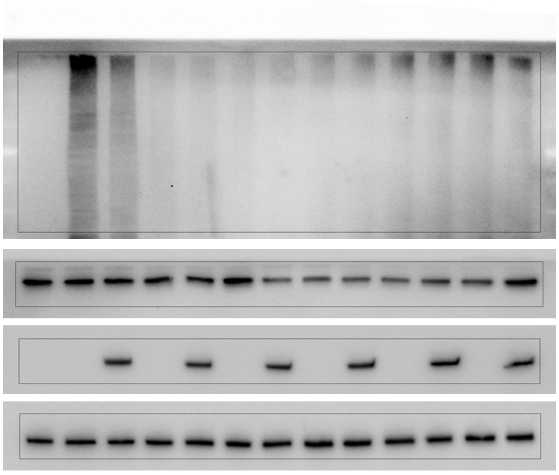

Uncropped blots Related to Figure 4

A

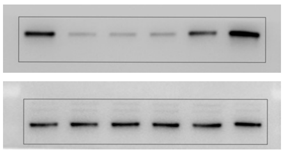

Uncropped blots Related to Figure 5

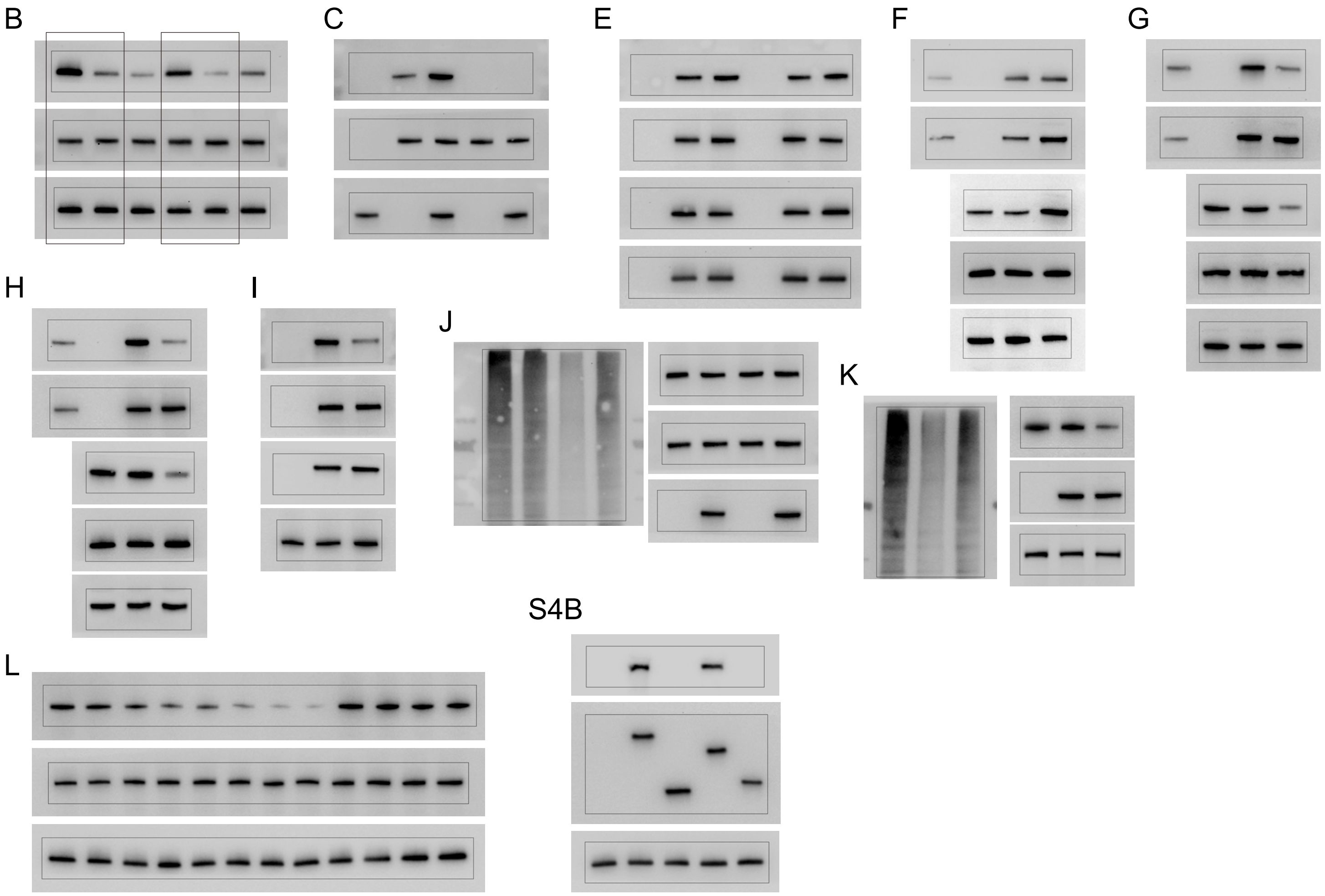

Uncropped blots Related to Figure 6

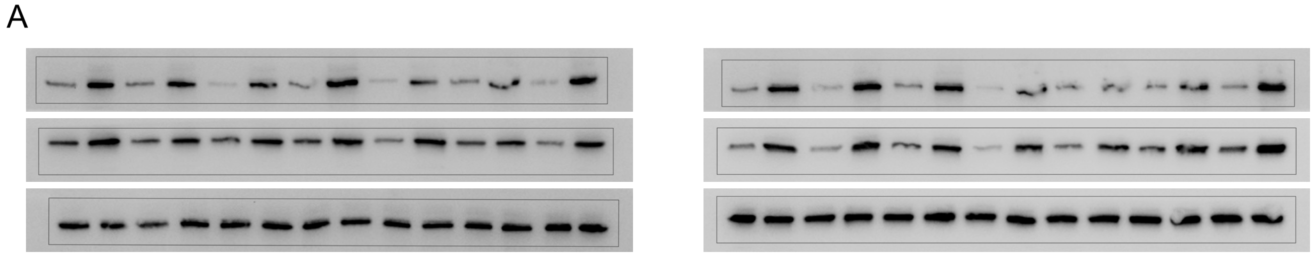

Supplement: Supplementary file 8 — Original Data File [file 41419_2023_6304_MOESM8_ESM.pdf]
